# Identification of Key Genes for Alcoholic Hepatitis Using Integrated Network Analysis of Differential lncRNA and Gene Expression

**DOI:** 10.3390/ijms26136104

**Published:** 2025-06-25

**Authors:** Bihuan Hu, Hui Xia, Peixuan Tian, Xinbao Li, Yu Yang, Zixuan Zhu, Yajie Zhou, Wang Liao, Shoakang Wang, Ligang Yang, Guiju Sun, Jing Sui

**Affiliations:** 1 Key Laboratory of Environmental Medicine and Engineering of Ministry of Education, Department of Nutrition and Food Hygiene, School of Public Health, Southeast University, Nanjing 210009, China; bihuanhu1010@163.com (B.H.); 220244286@seu.edu.cn (P.T.); 213204061@seu.edu.cn (X.L.); 220244291@seu.edu.cn (Y.Y.); zxzhu1002@163.com (Z.Z.); 13813996366@139.com (Y.Z.); wangliao@seu.edu.cn (W.L.); shaokangwang@seu.edu.cn (S.W.); yangligang2012@163.com (L.Y.); gjsun@seu.edu.cn (G.S.); 2 Research Institute for Environment and Health, Nanjing University of Information Science and Technology, Nanjing 210044, China

**Keywords:** alcoholic hepatitis, mRNAs, lncRNAs, hub gene analysis

## Abstract

Alcoholic liver disease (ALD) is a type of liver disease with complex pathogenic factors. In 2019, alcohol caused 11 million life-years to be lost globally, and the mortality rate has continued to rise. This study aims to explore the exclusive gene profile of AH and construct an mRNA-lncRNA regulatory network through an integrative analysis and database validation to reveal potential key biomarkers. We obtained expression data for alcoholic hepatitis from the GEO database; screened differentially expressed genes (DEGs) through a weighted gene co-expression network analysis (WGCNA); conducted a GO&KEGG analysis; and focused on the enrichment pathways for the top 20 genes. Hub genes were selected using cytoHubba and MCODE to construct the mRNA-lncRNA regulatory network, and key genes were confirmed using GSE167308 and GSE28619. We obtained 2552 differentially expressed mRNAs and 555 differentially expressed lncRNAs from three databases. Differentially expressed genes are mainly involved in pathways such as lipid metabolism disorders, complement activation, the activation of cancer-related pathways, the excessive activation of inflammatory immunity, and the initiation of cell adhesion and fibrosis. Based on the hub gene analysis, we screened out 43 key genes. By constructing the key mRNA-lncRNA–pathway network, we identified 12 mRNAs (AQP1, ELOVL7, ITPR3, KRT19, KRT23, LAMC2, MMP7, PROM1, SPINT1, STK39, TNFRSF21, and VTCN1) and 14 lncRNAs that play an important role in the occurrence and development of alcoholic hepatitis. To sum up, this article mainly expounds upon the key genes in the occurrence and development of alcoholic hepatitis. The key genes are mainly concentrated within signaling pathways such as metabolic pathways, fatty acid metabolism, and cancer pathways. Twelve differentially expressed mRNAs in the co-expression network can be used as biomarkers and intervention targets for the diagnosis and treatment of alcoholic hepatitis.

## 1. Introduction

Alcoholic fatty liver disease is a harmful lesion of the liver caused by long-term heavy drinking. It initially manifests as alcoholic fatty liver disease (AFLD) and gradually develops into alcoholic hepatitis (AH) with the accumulation of inflammation. Under the influence of persistent chronic liver damage, alcoholic hepatitis may progress to liver fibrosis, cirrhosis, and, in severe cases, hepatocellular carcinoma (HCC) [1]. If left untreated, it can lead to liver failure and become life-threatening. Alcohol is also a major cause of cirrhosis worldwide, with approximately 60% of cirrhosis cases in Europe, North America, and Latin America being caused by alcohol [2]. Excessive drinking has also been associated with a 260-fold increased risk of alcohol-related death and a 5.1-fold increased risk of death from cancer [3]. Due to the poor prognosis of alcoholic liver disease and the limitations of therapeutic drugs, early diagnosis and early intervention are essential to improving the clinical outcomes in people with alcoholic liver disease, and understanding the molecular mechanisms underlying alcoholic hepatitis is essential for identifying biomarkers for early diagnosis, as well as finding effective drugs.

Long non-coding RNAs (lncRNAs) are non-coding RNAs that are longer than 200 nucleotides [4]. LncRNAs have very important regulatory functions, such as dose compensation effects, epigenetic regulation, cell cycle regulation, and cell differentiation regulation, and are involved in various biological processes and pathways [5,6,7]. Studies have shown that LncRNA is closely related to the occurrence and development of various diseases [7,8,9,10]. Compared with messenger RNA (mRNA), the expression pattern for long non-coding RNA is more limited, and it plays a role in cell lineage specificity [11]. This characteristic makes it play an important role in cell life activities. LncRNAs participate in the genomic fabric, cell structure, and gene expression through multiple interactions. These interactions include the chromatin structure, transcriptional regulation, splicing, protein translation, and localization [12]. The biological functions of lncRNA can be roughly summarized as follows: (1) controlling the chromatin structure; (2) enhancement effects; and (3) the formation of biomolecular agglomerates [13].

The occurrence and development of alcoholic hepatitis involve many aspects, among which lipid metabolism, apoptosis, inflammation, and other mechanisms play important roles [14]. Most studies have shown that lncRNAs can affect the expression and function of related genes in these fields through a variety of regulatory mechanisms, including cell proliferation, epigenetic regulation, transcriptional regulation, and post-transcriptional regulation [15]. The role of lncRNAs in lipid metabolism involves several mechanisms. LncRNA MEG3 regulates the expression of NLRC5 and activates transcription factors such as NF-κB, thereby promoting hepatocyte steatosis and affecting lipid metabolism and decomposition [16]. LncRNA-AIRN can affect mitochondrial autophagy in alcoholic fatty liver disease by regulating the mTOR ubiquitination process [17]. In terms of apoptosis, lncRNAs may affect the NF-κB signaling pathway by activating ICAM-1, effectively inhibiting hepatocyte apoptosis and promoting hepatocyte proliferation [18]. LncRNA UCA1 is highly expressed in patients with alcoholic fatty liver disease and in ethanol-induced hepatocytes, which can inhibit the expression of miR-214 and promote the abnormal proliferation and invasion of hepatocytes [19]. In addition, other studies have shown that the long-chain non-coding RNA HOTAIR may regulate the proliferation of hepatic stellate cells by regulating the miR-148a-3p/S1PR1 axis, thereby alleviating the liver damage caused by alcoholic hepatitis [20,21]. In terms of inflammation, LncRNA1700020I14Rik can promote liver cell damage in AH mice by inhibiting miR-1’s activation of AKR10B137/Erk signaling [22]. Ethanol can affect the biological activity of extracellular vesicles in the endothelial cells by regulating the expression of lncRNA (especially HOTAIR and MALAT1), thus affecting the inflammatory symptoms of alcoholic hepatitis [23]. Therefore, we can also detect long non-coding RNAs in body fluids as a potential diagnostic tool and a therapeutic target for patients with fatty liver steatosis and alcoholic liver disease [24].

However, there are few studies on the relationship between mRNAs and lncRNAs in alcoholic hepatitis. To explore the mRNA-lncRNA co-expression relationships in alcoholic hepatitis, we systematically searched the GEO database and identified three datasets associated with alcoholic hepatitis expression profiles. Subsequently, we conducted a weighted gene co-expression network analysis (WGCNA) and hub gene identification. A functional enrichment analysis of the key genes was performed using Gene Ontology (GO) and Kyoto Encyclopedia of Genes and Genomes (KEGG). These findings were validated further using two independent datasets to elucidate the critical genes involved in the diagnosis of alcoholic hepatitis. This provides a novel target for the prevention and diagnosis of alcoholic hepatitis.

## 2. Results

### 2.1. Identification and Cluster Analysis of Differential Genes in Alcoholic Hepatitis

**Figure 1:**
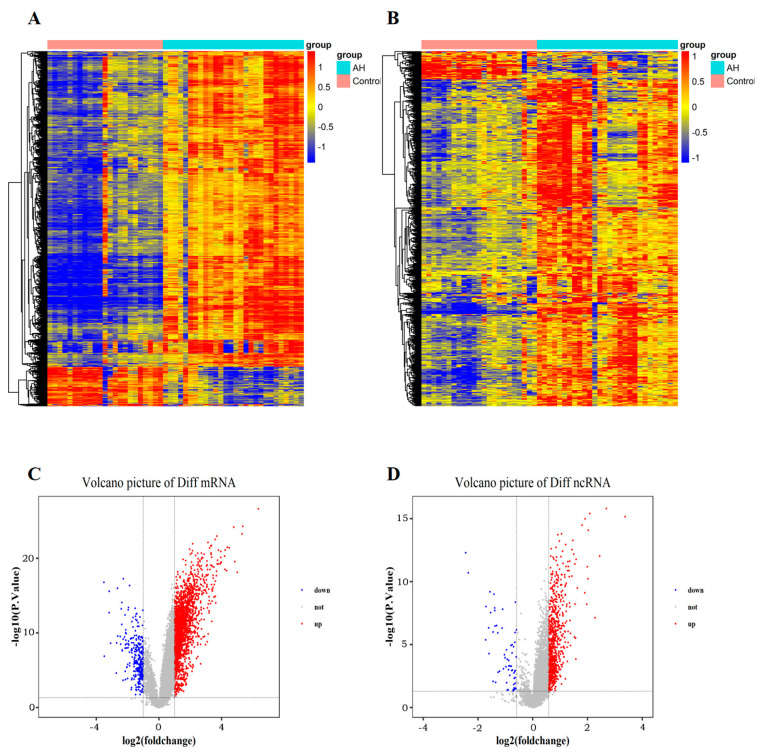
Differential expression analysis of mRNAs and lncRNAs in alcoholic hepatitis. (**A**,**C**) mRNAs; (**B**,**D**) lncRNAs.

Under the set conditions (an FC > 2 (mRNAs) or an FC > 1.5 (lncRNAs), *p* < 0.05, a false discovery rate (FDR) < 0.05), a total of 2552 mRNAs and 555 lncRNAs were detected. Compared with the normal control group, 2270 mRNAs were upregulated and 282 mRNAs were downregulated in alcoholic hepatitis. A total of 511 lncRNAs showed an upward trend, and 44 lncRNAs showed a downward trend. The differential expression analysis of these mRNAs and lncRNAs could be obtained as follows (Figure 1):

### 2.2. The GO and KEGG Pathway Analysis

**Figure 2:**
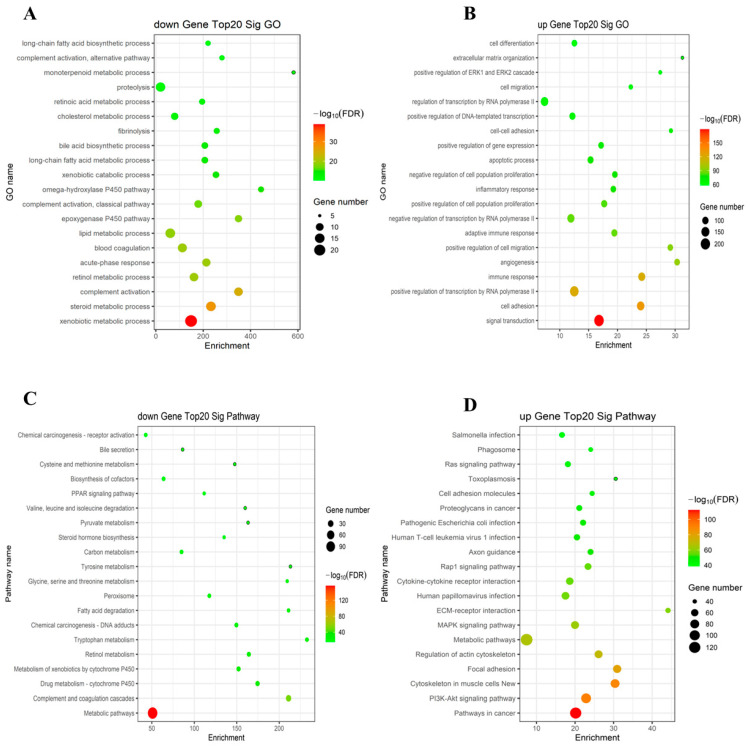
A Gene Ontology (GO) analysis and a Kyoto Encyclopedia of Genes and Genomes (KEGG) pathway enrichment analysis of the top 20 differentially expressed mRNAs. (**A**) The GO analysis of downregulated genes. (**B**) The GO analysis of upregulated genes. (**C**) Downregulated KEGG pathways of gene enrichment. (**D**) Upregulated KEGG pathways of gene enrichment (the size of the spots corresponds to the quantity of mRNAs, and the color of the spots represents the *p*-value).

To analyze the functions of the differentially expressed genes further, we detected the top 20 significantly differentially expressed genes (upregulated or downregulated) through a GO analysis and a KEGG pathway enrichment analysis (Figure 2). We found that the downregulated genes were mainly concentrated in processes such as lipid metabolism, complement activation, and exogenous substance metabolism, which suggested that excessive alcohol intake impairs the body’s lipid metabolism, immune response, and the liver’s detoxification function (Figure 2A). The upregulated genes are mainly concentrated in biological processes such as cell proliferation and migration, extracellular matrix (ECM) recombination, immune and inflammatory responses, and transcriptional regulation (Figure 2B). This suggests that upregulation of the inflammation–immune storm and fibrosis genes plays an important role in the pathogenesis of alcoholic hepatitis. Subsequently, we conducted an analysis of the key differential gene KEGG pathways and found that the downregulated genes were mainly concentrated in lipid metabolism disorders, complement activation, and impaired detoxification functions (Figure 2C). Upregulated genes are mainly associated with the activation of cancer-related pathways, the excessive activation of inflammatory immunity, and the initiation of cell adhesion and fibrosis (Figure 2D). This suggests that these differentially expressed genes play an important role in alcoholic hepatitis by influencing these key pathways.

### 2.3. Construction of the Co-Expression of Differential Genes and the Hub Gene Analysis

**Figure 3:**
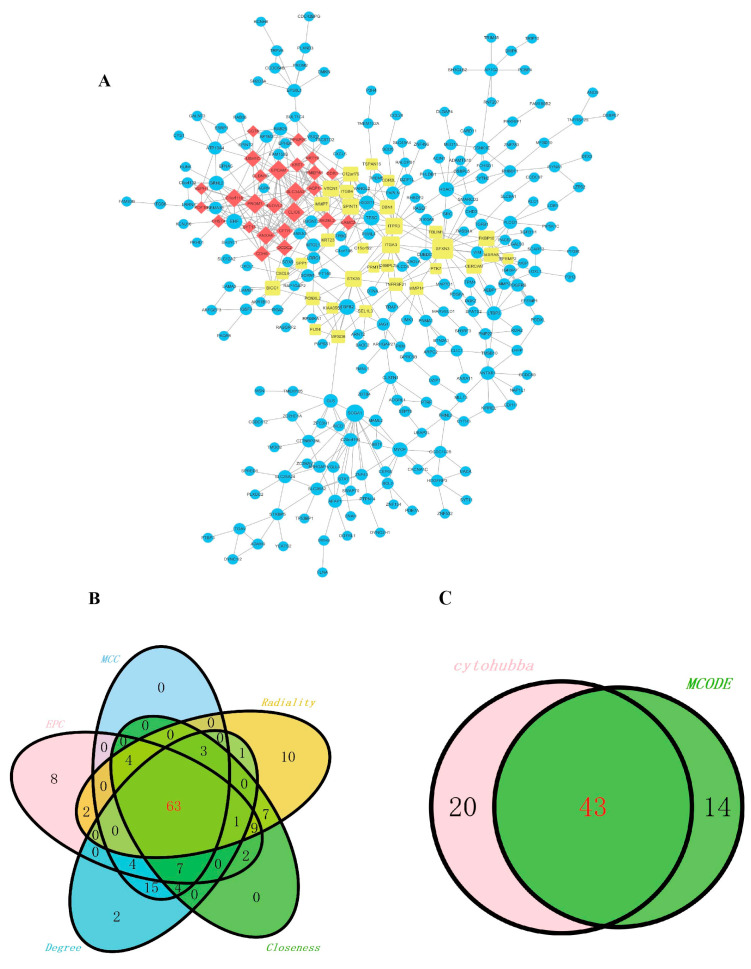
Analysis of hub genes. (**A**) mRNA co-expression network (**B**) Venn diagram of cytoHubba. (**C**) Venn diagram of cytoHubba and MCODE.

After screening for differential genes, a network of differential gene interactions was constructed, as shown in Figure 3A. Subsequently, the cytoHubba and MCODE plugins in Cytoscape were used to conduct the network topology analysis and the node center character analysis on the gene interaction network and to sequence the genes. Research has found that the degree of a protein is directly related to the importance of its genes. The cytoHubba plugin identifies the key nodes in the gene interaction network, and it is seen that there are 63 key genes (Figure 3B). Using the analysis of the MCODE plugin, 57 key genes were discovered. Through the intersection of the Venn diagrams, we eventually discovered 43 key genes (Figure 3C).

### 2.4. Construction of the mRNA-LncRNA Co-Expression Network

**Figure 4:**
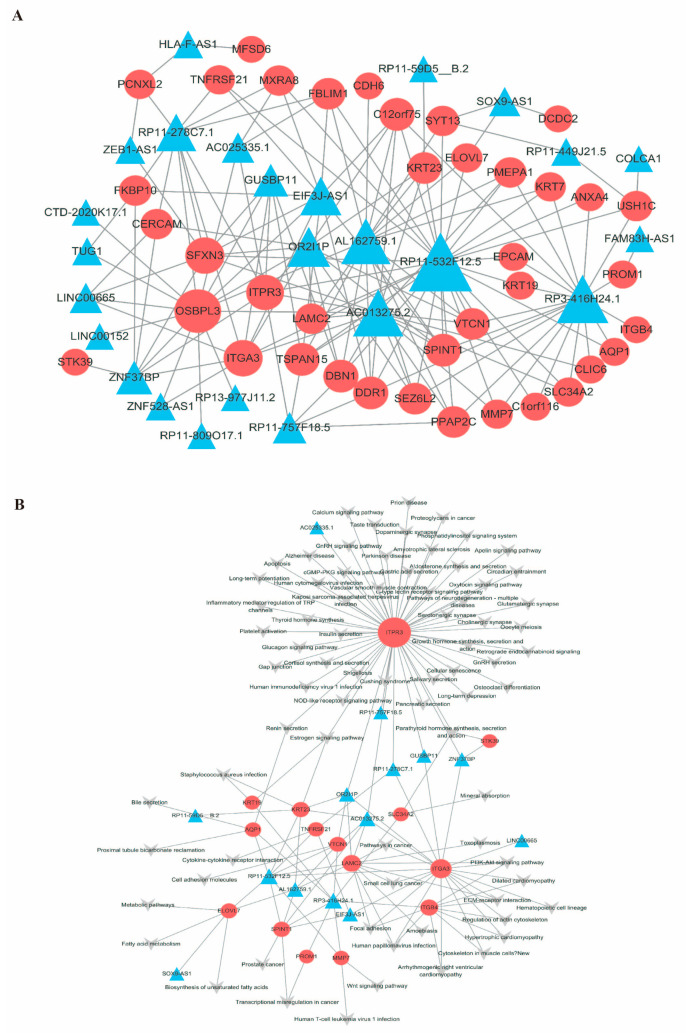
mRNA-lncRNA co-expression network (**A**) mRNA-lncRNA co-expression network. (**B**) mRNA-lncRNA co-expression pathways of hub genes. Circular nodes represent mRNA, triangular nodes represent lncRNA, and arrow nodes represent pathways.

The mRNA-lncRNA co-expression network was constructed to detect the functional mechanism of lncRNAs in key modules (Figure 4A). We found that 37 mRNAs and 22 lncRNAs regulated each other; 1 lncRNA could regulate multiple mRNAs. In addition, one mRNA could also regulate multiple lncRNAs. This shows that there is a complex regulatory relationship between mRNAs and lncRNAs.

### 2.5. Construction of the mRNA-lncRNA–Pathway Co-Expression Network

According to the interaction network of mRNAs-lncRNAs and mRNA pathways, we obtained the mRNA-lncRNA–pathway network (Figure 4B). We identified 15 mRNAs and 14 lncRNAs in the mRNA-lncRNA–pathway network, and these genes were upregulated in patients with alcoholic hepatitis. In the module, RP11-532F12.5 (lncRNA) was connected with nine mRNAs (AQP1, ELOVL7, SPINT1, KRT23, TNFRSF21, VTCN1, LAMC2, ITGA3, and MMP7) and concentrated in metabolic pathways, fatty acid metabolism, transcriptional disorders in cancer, the Wnt signaling pathway, human T-cell leukemia virus 1 infection, the PI3K-Akt signaling pathway, and other signaling pathways. RP3-416H24.1 was connected with seven mRNAs (SPINT1, PROM1, MMP7, VTCN1, AQP1, KRT19, SLC34A2, and ITGB4) and enriched in prostate cancer, transcriptional disorders in cancer, the Wnt signaling pathway, human T-cell leukemia virus 1 infection, ECM–receptor interaction, hypertrophic cardiomyopathy, staphylococcus aureus infection, bile secretion, metabolic pathways, fatty acid metabolism, and other signaling pathways.

### 2.6. Verification of Pivotal mRNAs with Diagnostic Value

**Figure 5:**
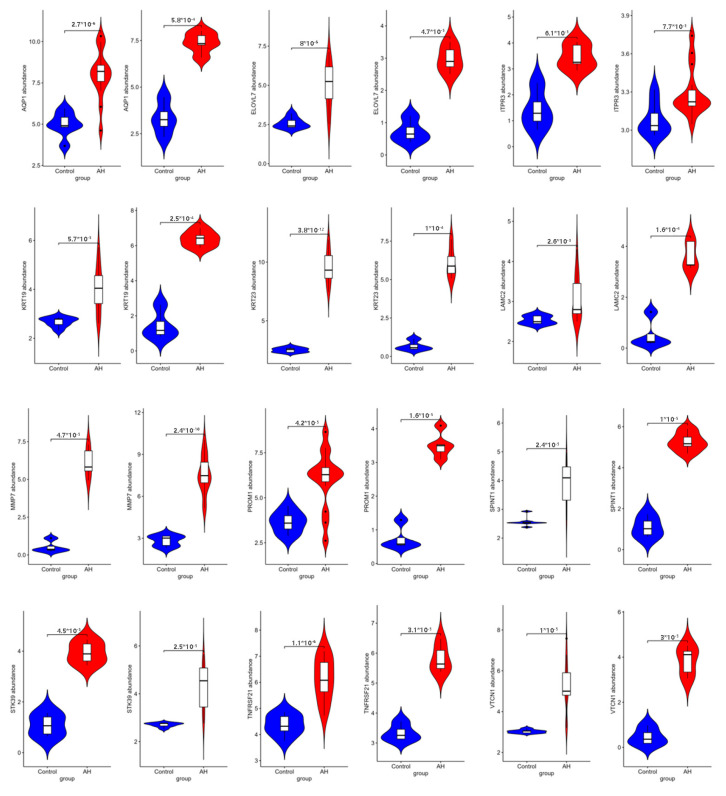
The differential expression of GSE167308 and GSE28619 in alcoholic hepatitis diagnosis (12 mRNAs (AQP1, ELOVL7, ITPR3, KRT19, KRT23, LAMC2, MMP7, PROM1, SPINT1, STK39, TNFRSF21, and VTCN1) show an upregulation trend in the development of alcoholic hepatitis).

We used GSE167308 and GSE28619 to verify the 15 mRNAs found above. Twelve genes were found (*AQP1*, *ELOVL7*, *ITPR3*, *KRT19*, *KRT23*, *LAMC2*, *MMP7*, *PROM1*, *SPINT1*, *STK39*, *TNFRSF21*, and *VTCN1*) that showed an upregulation trend in the development of alcoholic hepatitis, which was consistent the previous analysis (Figure 5). This suggests that these 12 genes may have important implications for the diagnosis of alcoholic hepatitis. However, the expressions of SLC34A2, ITGA3, and ITGB4 were all upregulated in the GSE28619 dataset, while there was no significant difference in the GSE167308 dataset.

## 3. Discussion

Alcoholic liver disease is one of the most common liver diseases worldwide. Alcoholic hepatitis is an acute liver injury in people who consume excessive amounts of alcohol, characterized by acute or chronic liver failure, which is associated with a rapid decline in liver synthesis, resulting in increased mortality. Studies have shown that the average 30-day mortality rate for patients with severe alcoholic hepatitis can be as high as 17–50% [25]. In recent years, alcoholic hepatitis has increased in young people and women, which is associated with multiple comorbidities (obesity, non-alcoholic fatty liver disease) and the side effects of the pandemic [3,26]. The incidence and severity associated with alcohol use disorders are currently on the rise. The current treatments for alcoholic hepatitis focus on abstinence from alcohol and nutritional interventions, such as vitamin, mineral, and glucocorticoid supplementation [27,28]. Alcohol abstinence is an effective treatment, but it is difficult to achieve in most people with alcoholic hepatitis due to alcohol dependence, and sudden withdrawal can cause alcohol withdrawal syndrome, which in severe cases can lead to epilepsy (mainly because the suppressed central nervous system is overactivated in an alcohol-free environment) [29]. Liver biopsy remains the gold standard for diagnosing alcoholic hepatitis, but it is not widely used due to its traumatic nature [30]. At present, the diagnosis of alcoholic hepatitis is mainly assessed by combining clinical symptoms and laboratory data, but there is still great heterogeneity in diagnoses based on these data alone [31]. Therefore, the search for effective and non-invasive biomarkers for the diagnosis of alcoholic hepatitis, as well as understanding the pathogenesis of alcoholic liver disease and potential therapeutic and diagnostic targets, is also a current research priority.

Most studies have shown that the occurrence and development of alcoholic hepatitis are related to a variety of signaling pathways, and alcohol-mediated epigenetic modifications are related to changes in mRNAs and lncRNAs [32]. In this study, we analyzed mRNAs and lncRNAs associated with alcoholic hepatitis through bioinformatics, searched for differentially expressed mRNAs, constructed an mRNA-lncRNA co-expression network, and verified differentially expressed mRNAs through multiple datasets. By analyzing the mRNAs and lncRNAs in normal liver tissue and liver tissue samples from alcoholic hepatitis patients, the mRNA-lncRNA co-expression network was constructed to identify the potential pathogenesis of AH. The GSE143318, GSE142530, and GSE155907 datasets were analyzed. Compared with the control group without alcoholic hepatitis, 2270 mRNAs were upregulated in the hepatitis patients, and 282 mRNAs were downregulated. A total of 511 lncRNAs showed an upward trend, and 44 lncRNAs showed a downward trend. According to the GO and KEGG analyses, the expression of differential genes is mainly concentrated in metabolic pathways, fatty acid metabolism, and pathways in cancer. To identify the key genes in pathogenesis, we identified 43 key genes through the hub gene analysis. In addition, we also constructed the mRNA-lncRNA–pathway co-expression network and found that 15 mRNAs and 14 lncRNAs were significantly upregulated, and the signaling pathways involved in 14 lncRNAs were also revealed.

The liver is one of the most dynamic organs in the human body and is closely related to various metabolic processes. The liver plays a key role in fatty acid metabolism, bile acid metabolism, gluconeogenesis, liver glycogen synthesis and catabolism, glycolysis, plasma protein metabolism, the metabolism for various vitamins, drug/exogenous metabolism, and detoxification [33]. Compared with the normal liver, the expression of metabolism-related genes in the livers of alcoholic hepatitis patients was significantly downregulated, which may be because the toxicity of the metabolites in alcohol pressures metabolic processes in the body [34]. Due to the development of antiviral drugs, the incidence of liver cancer caused by viruses has declined, but the incidence of alcohol abuse and metabolic-related liver cancer is on the rise [35,36]. The analysis of the data showed that genes associated with cancer were highly expressed. Population studies have shown that patients with alcoholic liver disease have a higher incidence of cancer (both full cancer and liver cancer) [37]. The PI3K-Akt signaling pathway can promote cell survival, cell growth, and cell cycle progression, and dysregulation of signal transduction may promote the development of cancer [38,39]. Compared with the group without AH, genes related to the PI3K-Akt signaling pathway were upregulated in the patients with alcoholic hepatitis. Studies have shown that activation of the PI3K-Akt signaling pathway increases cancer’s aggressiveness, and AKT pathway activation has been shown to be a significant risk factor for early recurrence and poor prognosis in patients with liver cancer [40]. PI3K is highly expressed in the liver tissues of HCC patients and is positively correlated with a poor prognosis in HCC patients [39,41]. Therefore, the PI3K-Akt signaling pathway plays an important role in the pathogenesis of alcoholic hepatitis.

According to the mRNA-lncRNA–pathway co-expression network, compared with the group without AH, the genetic differences in the alcoholic hepatitis group were mainly concentrated in metabolic pathways, fatty acid metabolism, transcriptional disorders in cancer, the PI3K-Akt signaling pathway, the Wnt signaling pathway, human T-cell leukemia virus 1 infection, and other signaling pathways, indicating that alcoholic hepatitis is a multifactorial disease. LncRNAs play an important role in the development of alcoholic hepatitis. RP11-532F12.5 (lncRNA) is linked to nine mRNAs (ITGA3, LAMC2, KRT23, ELOVL7, SPINT1, AQP1, TNFRSF21, VTCN1, and MMP7). ITGA3 is closely associated with a poor prognosis in various cancers, and its expression is upregulated in most tumor tissues. Laminin are key proteins in the basal layer that influence normal and transformed cell differentiation, migration, and adhesion, as well as phenotype and survival [42]. LAMC2 (a laminin component) may contribute to cancer development by interacting with receptors on cells [43]. Gene expression profiles show that KRT23 is moderately highly expressed in steatohepatitis, suggesting that KRT23 may be a potential biomarker for alcoholic hepatitis and a marker for predicting HCC progression [44]. The ELOVL7 gene is closely related to lipid metabolism and plays an important role in the synthesis of long-chain unsaturated fatty acids [45]. SPINT1 also plays an important role in various malignant cancers [46].

According to the co-expression network, lncRNAs play an important role in the occurrence and development of alcoholic hepatitis. RP11-532F12.5 interacts with these mRNAs and plays an important role in the pathogenesis of alcoholic hepatitis. Studies have shown that the abnormal regulation of the RP11 gene family is highly correlated with the occurrence of most cancers [47,48,49]. For example, the expression of RP11-295GG20.2 is significantly upregulated in liver cancer tissues, and its high expression is closely related to a poor prognosis in liver cancer patients [47]. RP11-323N12.5 is the lncRNA with the highest degree of upregulation in human gastric cancer. It promotes YAP1 transcription by binding to c-MYC in the YAP1 promoter in gastric cancer and T-cells [48]. The expression of RP11-532F12.5 significantly increases in patients with alcoholic hepatitis. It may regulate the cancer pathway through the co-expression of the LAMC2 gene and thereby affect the development process of alcoholic hepatitis. Equally, it may upregulate metabolic pathways and fatty acid metabolism by regulating other related mRNAs (AQP1, ELOVL7, SPINT1, KRT23, TNFRSF21, VTCN1, ITGA3, and MMP7). Signaling pathways such as metabolism and transcriptional misregulation in cancer play significant roles in the progression of alcoholic hepatitis.

In addition, 12 genes (*AQP1*, *ELOVL7*, *ITPR3*, *KRT19*, *KRT23*, *LAMC2*, *MMP7*, *PROM1*, *SPINT1*, *STK39*, *TNFRSF21*, and *VTCN1*) are expected to become new diagnostic markers, as they were verified using the GSE167308 and GSE28619 databases, and all 12 genes were significantly upregulated in alcoholic hepatitis.

## 4. Materials and Methods

### 4.1. The Data Collection

**Figure 6:**
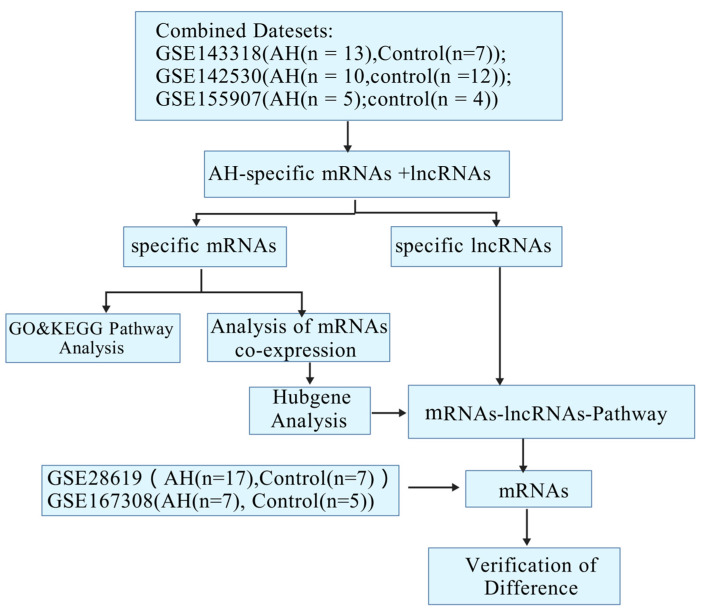
The flow chart for this study.

We downloaded alcoholic hepatitis expression data from the GEO database dataset (GSE143318 GSE142530, GSE155907) and then processed them into standardized data for the subsequent analysis. GSE143318 contained 13 alcoholic hepatitis samples and 7 normal control samples; GSE142530 contained 10 alcoholic hepatitis samples and 12 normal control samples; and GSE155907 contained 5 alcoholic hepatitis samples and 4 normal control samples, yielding a total of 28 alcoholic hepatitis samples and 23 normal control samples. The R software package was used to analyze lncRNAs and mRNAs that were significantly differentially expressed between the control and exposed groups according to the screening criteria (multiple changes > 2 or >1.5, *p* < 0.05, a false discovery rate (FDR) < 0.05). The flow chart for this study is shown in Figure 6.

### 4.2. Gene Ontology and Pathway Enrichment Analysis

Functional annotations of the mRNAs in each module were analyzed using the Gene Ontology (GO) database. The Kyoto Encyclopedia of Genes and Genomes (KEGG) database is a public pathway database used to perform pathway enrichment analyses. Fisher’s exact test and a multiple comparison test were used to calculate the *p*-values and FDRs. *p* < 0.05 and an FDR < 0.05 indicated significant differences.

### 4.3. A Co-Expression Analysis of Differentially Expressed Genes

To explore the interaction between each key gene, we constructed a gene interaction network using key genes, examined the connections between genes, and then identified the key genes. Circles, squares, and triangles all represent genes, and straight lines represent the regulatory relationships existing for genes. The size of a circle represents the degree value, that is, the number of relationships between a certain gene in the network and its surrounding genes. The larger the degree, the more genes it has an interaction relationship with.

### 4.4. The Hub Gene Analysis

After constructing the co-expression network of differentially expressed genes, we used the cytoHubba plugin to conduct a network topology analysis and a node center character analysis. Each gene was assigned values and ranked through the topological network to discover the key genes and sub-networks within it. CytoHubba is a plugin in the Cytoscape software (3.10.2) for identifying hub nodes that provides five analytical algorithms for computing the hub genes in protein interaction network diagrams. It includes the degree, the edge permeability component (EPC), the maximum neighborhood component (MNC), the density of the maximum neighborhood component (DMNC), the maximum group centrality (MCC), and centrality based on the shortest path. MCODE (Molecular COmplex Detection) can be used to discover tightly connected regions in gene interaction networks, which may represent molecular complexes. According to the given parameters, the dense regions are separated.

The central gene is defined by higher connectivity in the module. Central genes with high connectivity are upstream regulators, while low-connectivity genes are downstream regulators. The co-expression relationships between the hub genes were calculated, and mRNA-lncRNA co-expression and mRNA co-expression were selected for constructing the co-expression network.

### 4.5. Construction of the lncRNA-mRNA Pathway Co-Expression Network

To construct the lncRNA-mRNA pathway network, the regulatory relationships between the lncRNAs and mRNAs were examined, as were the important pathways involved in the regulation of key mRNAs. The main purpose of this study is to reveal the signaling pathways of the key genes regulated by lncRNA to predict potential diseases related to lncRNA.

### 4.6. Verification of Hub Genes

Two datasets, GSE167308 and GSE28619, were used to verify the difference in the expression of key genes between patients with and without alcoholic hepatitis through a differential expression validation analysis.

### 4.7. Data Analysis

R software (version 4.1.1) and SPSS (version 21.0) were used for the statistical analysis. Standardized datasets were statistically compared using Student’s *t* test or a one-way ANOVA. *p* < 0.05 was considered significant.

## 5. Conclusions

In summary, this paper mainly describes the key genes in the occurrence and development of alcoholic hepatitis. Twelve mRNAs (AQP1, ELOVL7, ITPR3, KRT19, KRT23, LAMC2, MMP7, PROM1, SPINT1, STK39, TNFRSF21, and VTCN1) and fourteen lncRNAs play an important role in the occurrence and development of alcoholic hepatitis, and database validation shows that these genes are significantly upregulated in patients with alcoholic hepatitis, which provides further directions for the study of useful diagnostic markers and molecular mechanisms in alcoholic hepatitis. However, this study still has some limitations. Further studies require larger samples of alcoholic hepatitis patients, and more experiments are needed to explore the biological functions of the key lncRNAs.

## Data Availability

The original data presented in the study are openly available in GEO datasets at https://www.ncbi.nlm.nih.gov/geo/query/acc.cgi?acc=GSE143318; https://www.ncbi.nlm.nih.gov/geo/query/acc.cgi?acc=GSE142530; https://www.ncbi.nlm.nih.gov/geo/query/acc.cgi?acc=GSE155907; https://www.ncbi.nlm.nih.gov/geo/query/acc.cgi?acc=GSE28619; https://www.ncbi.nlm.nih.gov/geo/query/acc.cgi?acc=GSE167308 (accessed on 19 June 2025).
